# Prophylactic mastectomy and occult malignancy: Surgical and imaging considerations

**DOI:** 10.1002/jso.27088

**Published:** 2022-09-07

**Authors:** Jessica L. Thompson, Brandy R. Sinco, Rachel L. McCaffrey, Alfred E. Chang, Michael S. Sabel, Lesly A. Dossett, Tasha M. Hughes, Jacqueline S. Jeruss

**Affiliations:** ^1^ Department of Surgery, Division of Surgical Oncology University of Michigan Ann Arbor Michigan USA; ^2^ Center for Healthcare Outcomes and Policy University of Michigan Ann Arbor Michigan USA; ^3^ Department of Surgery Vanderbilt University Nashville Tennessee USA

**Keywords:** breast cancer, occult malignancy, preoperative breast imaging, prophylactic mastectomy, sentinel lymph node biopsy

## Abstract

**Background:**

Sentinel node biopsy (SLNB) is not routinely recommended for patients undergoing prophylactic mastectomy (PM), yet omission remains a subject of debate among surgeons. A modern patient cohort was examined to determine occult malignancy (OM) incidence within PM specimens to reinforce current recommendations.

**Methods:**

All PM performed over a 5‐year period were retrospectively identified, including women with unilateral breast cancer who underwent synchronous or delayed contralateral PM or women with elevated cancer risk who underwent bilateral PM.

**Results:**

The study population included 772 patients (598 CPM, 174 BPM) with a total of 39 OM identified: 17 invasive cancers (14 CPM, 3 BPM) and 22 DCIS (19 CPM, 3 BPM). Of the 86 patients for whom SLNB was selectively performed, 1 micrometastasis was identified. In the CPM cohort, risk of OM increased with age, presence of LCIS of either breast, or presence of a non‐*BRCA* high‐penetrance gene mutation, while preoperative magnetic resonance imaging was associated with lower likelihood of OM.

**Conclusions:**

Given the low incidence of invasive OM in this updated series, routine SLNB is of low value for patients undergoing PM. For patients with indeterminate radiographic findings, discordant preoperative biopsies, LCIS, or non‐*BRCA* high‐penetrance gene mutations, selective SLNB implementation could be considered.

**Abbreviations**
ADH: atypical ductal hyperplasia
AIC: Akaike Information Criteria
ALH: atypical lobular hyperplasia
ASBrS: American Society of Breast Surgeons
BI‐RADS: breast‐imaging reporting and data system
BPM: bilateral prophylactic mastectomy
BSO: bilateral salpingo‐oophorectomy
CPM: contralateral prophylactic mastectomy
DCIS: ductal carcinoma in‐situ
ER: estrogen receptor
HER2: human epidermal growth factor receptor 2
IDC: invasive ductal carcinoma
IDP: intraductal papilloma
ILC: invasive lobular carcinoma
IMC: invasive mammary carcinoma
LCIS: lobular carcinoma in‐situ
MRI: magnetic resonance imaging
OM: occult malignancy
OR: odds ratio
PM: prophylactic mastectomy
PR: progesterone receptor
SD: standard deviation
SLNB: sentinel lymph node biopsy
SPIO: superparamagnetic iron oxide nanoparticles
VUS: variant of unknown significance

## 1 INTRODUCTION

Prophylactic mastectomy (PM) refers to the surgical removal of one or both breasts in the absence of malignancy. Bilateral prophylactic mastectomy (BPM) is a consideration for individuals with an elevated lifetime risk of developing breast cancer, such as those with a genetic predisposition, atypical proliferative changes within the breast tissue, prior chest wall irradiation, and high breast density. Patients who are planning to undergo, or who may have previously undergone, therapeutic mastectomy for unilateral breast cancer may elect to pursue contralateral prophylactic mastectomy (CPM).

Rates of CPM in the United States have been on the rise for the past several decades.^1^, ^2^, ^3^ While this trend is presumptively multifactorial, it is surmised to be largely patient‐driven.^3^, ^4^, ^5^ Escalating utilization of germline genetic testing together with improved access to chest wall reconstruction options have contributed to the increasing interest in PM.^5^, ^6^, ^7^, ^8^, ^9^, ^10^ Additionally, preoperative magnetic resonance imaging (MRI) has been associated with higher CPM receipt.^4^, ^5^, ^11^

In preparation for surgery, breast imaging, including mammography, ultrasound, and/or MRI, is routinely obtained to assess for suspicious findings that may warrant additional preoperative workup, diagnosis, and treatment. Despite evolving advances in breast imaging diagnostic capabilities, a percentage of patients undergoing PM will have incidental cancer detected at the time of final surgical pathologic examination. Prior studies investigating occult malignancy (OM) of the breast have recorded rates ranging from 3% to 10%.^12^, ^13^, ^14^, ^15^, ^16^, ^17^, ^18^, ^19^, ^20^

In the prophylactic setting, in the absence of abnormal radiographic findings, there is not a clear indication for axillary lymph node sampling. In 2016, the American Society of Breast Surgeons (ASBrS) advised against the routine completion of sentinel lymph node biopsy (SLNB) for CPM.^21^ Nonetheless, this recommendation has not been universally integrated into surgical practice patterns.^22^

While the prevalence of PM may remain persistently high, there is an opportunity for surgeons to reduce axillary overtreatment in the prophylactic setting. We sought to analyze a modern cohort of BPM and CPM patients with the aim of critically evaluating clinical and pathological factors associated with OM that could be utilized to inform decision making for the selective implementation of SLNB.

## 2 MATERIALS AND METHODS

### 2.1 Patient selection

A study protocol was approved by the Institutional Review Board. A prospectively maintained surgical database was utilized to retrospectively identify all PMs performed consecutively at our institution between October 1, 2016, and September 30, 2021. Criteria for study inclusion included (1) women with a personal history of unilateral breast cancer who underwent either synchronous or delayed contralateral PM (2) women with elevated breast cancer risk who underwent bilateral PM. Patients with a preoperative diagnosis of biopsy‐proven bilateral breast carcinoma within 12 months of the date of surgery and those who underwent chest masculinization operations were excluded. The study cohort was comprised of 772 patients, and a total of 946 PM specimens were analyzed.

### 2.2 Data collection

Data were collected retrospectively by reviewing clinic notes, operative reports, pathology records, and breast imaging documentation. Preoperative imaging was considered when performed within 1 year up to the day of surgery and included screening mammography, diagnostic mammography, breast ultrasound, and breast MRI. High‐risk lesions were defined as atypical ductal hyperplasia (ADH), atypical lobular hyperplasia (ALH), or lobular carcinoma in‐situ (LCIS). Occult malignancies were defined as ductal carcinoma in‐situ (DCIS) or infiltrating breast cancer identified incidentally during pathologic examination. Cases were classified by the highest‐risk lesion identified on pathologic assessment. Prophylactic SLNB was performed at the discretion of the operating surgeon. Given the prevalence of *BRCA* mutation carriers in the study population, *BRCA1* and *2* were analyzed separately from the other high‐penetrance genes, which included *PALB2, CDH1, TP53*, and PTEN.

### 2.3 Statistical analysis

Statistical significance was set at a *p* value of ≤0.05. Baseline descriptive statistics are presented in Table 1. To test for differences between the contralateral and bilateral groups, the *t*‐test was used for numerical variables that were approximately symmetric. Nonordinal categories were compared by the Pearson *χ* ^2^ test, provided that the expected cell counts were ≥5. The Fisher exact test was used when categorical variables had small cell sizes. The Cochran–Armitage trend test was used for ordinal categories, such as age group.

**Table 1:** Patient demographics, mean (SD) or *N* (%), *N* = 772

	Contralateral	Bilateral	*p* Value
	*N* = 598	*N* = 174	
Age (years)	50.4 (11.0)	43.4 (10.9)	<0.001^a^
Age categories			<0.001^b^
20–29	12 (2.0)	19 (10.9)	
30–39	84 (14.0)	42 (24.1)	
40–49	205 (34.3)	64 (36.8)	
50–59	158 (26.4)	36 (20.7)	
60–69	111 (18.6)	11 (6.3)	
70–79	28 (4.7)	2 (1.1)	
Menopausal status			0.665^c^
Premenopausal	281 (47.0)	85 (48.9)	
Postmenopausal	317 (53.0)	89 (51.1)	
Prior bilateral salpingo‐oophorectomy (BSO)	95 (15.9)	62 (35.6)	<0.001^c^
Genetics			<0.001^d^
*BRCA1/2*	70 (11.7)	95 (54.6)	<0.001^c^
High‐penetrance	16 (2.7)	22 (12.6)	<0.001^c^
Moderate‐penetrance	34 (5.7)	25 (14.4)	<0.001^c^
Variant of unknown significance (VUS)	84 (14.0)	3 (1.7)	<0.001^d^
Negative	233 (39.0)	23 (13.2)	<0.001^c^
No genetic testing	161 (26.9)	6 (3.4)	<0.001^c^
Preoperative MRI	231 (38.6)	141 (81.0)	<0.001^c^
Neoadjuvant chemotherapy	144 (29.7)		
Index cancer			
Invasive ductal	348 (58.2)		
Invasive lobular or mammary	89 (14.9)		
Other invasive	18 (3.0)		
Ductal carcinoma in‐situ	95 (15.9)		
Delayed PM/cancer treatment completed > 1 year before PM	48 (8.0)		
Index cancer receptor status			
ER‐positive	377 (68.8)		
PR‐positive	45 (8.2)		
HER2 neu positive	24 (4.4)		
HER2 neu negative	102 (18.6)		
Ipsilateral ADH/ALH	84 (14.0)	20 (11.5)	0.385^c^
Atypical lobular hyperplasia (ALH)	56 (9.4)	17 (9.8)	0.872^c^
Atypical ductal hyperplasia (ADH)	42 (7.0)	6 (3.4)	0.086^c^
History/presence of lobular carcinoma in‐situ	30 (5.0)	13 (7.5)	0.214^c^
Sentinel lymph node biopsy performed	60 (10.0)	26 (14.9)	0.070^c^
Reconstruction	404 (67.6)	156 (89.7)	<0.001^c^
Immediate reconstruction	373 (92.3)	154 (98.7)	0.002^d^
Delayed reconstruction	31 (7.7)	2 (1.3)	
Body mass index			n/a
Average	29.0 (6.9)	29.2 (6.9)	0.773^a^
Obesity category			0.636^c^
<30	256 (42.8)	78 (44.8)	
≥30	342 (57.2)	96 (55.2)	
Mantle field radiotherapy	10 (1.7)	1 (0.6)	0.471^d^
Imaging to surgery time (days)	92.4 (67.2)	111.3 (79.7)	0.005^a^
Categories for imaging^e^			0.002^b^
1–60 days	257 (43.0)	58 (33.3)	
61–120 days	174 (29.1)	46 (26.4)	
121–180 days	80 (13.4)	40 (23.0)	
181–240 days	59 (9.9)	11 (6.3)	
241–300 days	20 (3.3)	12 (6.9)	
301–365 days	3 (0.5)	5 (2.9)	
Not available	5 (0.8)	2 (1.1)	
MRI to surgery time (days)	102.8 (71.8)	135.3 (86.7)	<0.001^a^
Categories for Imaging^e^			
1–60 days	82 (13.7)	32 (18.4)	<0.001^b^
61–120 days	79 (13.2)	40 (23.0)	
121–180 days	27 (4.5)	35 (20.1)	
181–240 days	35 (5.9)	12 (6.9)	
241–300 days	7 (1.2)	12 (6.9)	
301–365 days	3 (0.5)	10 (5.7)	
Not available	365 (61.0)	33 (19.0)	
Occult malignancy	33 (5.5)	6 (1.7)	0.273^c^

Abbreviations: MRI, magnetic resonance imaging; SD, standard deviation.

^a^ *t*‐test.

^b^ Cochran–Armitage trend test.

^c^ Pearson *χ* ^2.^

^d^ Fisher exact test.

^e^ (241–300) and (301–365) combined into single category, not available ignored for *χ* ^2^ test.

Logistic regressions for “OM” were conducted separately for women who had contralateral and bilateral mastectomies. Due to OM being a rare event, the logistic regression models were estimated with Firth's bias‐reducing penalized likelihood method.^23^ First, the odds ratios were calculated for the individual predictors and the results are displayed in Table 2. Then, multivariable models were developed by including the variables that were significant at *p* < 0.1 in the univariable logistic regressions and selecting the combination of variables that minimized the Akaike Information Criteria (AIC).^24^ The *C* statistic was used to assess the fit of the logistic regression model with *C* ≥ 0.7 indicating a good fitting model.^25^

**Table 2:** Individual factors associated with occult malignancy finding in prophylactic mastectomy patients, OR (95% CI), *N* = 772.

*Predictor*	Contralateral, *N* = 598		Bilateral, *N* = 174	
	OR (95% CI)	*p* Value	OR (95% CI)	*p* Value
Age (years)	1.04 (1.01, 1.08)	0.008	1.02 (0.95, 1.09)	0.567
Age categories		0.078		0.496
20–29	Reference		Reference	
30–39	0.31 (0.07, 1.36)		0.57 (0.11, 2.92)	
40–49	0.75 (0.33, 1.73)		0.12 (0.01, 1.44)	
50–59	1.75 (0.82, 3.75)		1.64 (0.44, 6.06)	
60–69	1.08 (0.44, 2.64)		2.24 (0.39, 12.68)	
70–79	3.21 (1.14, 9.07)		3.13 (0.13, 75.20)	
Postmenopausal	1.56 (0.76, 3.20)	0.222	1.76 (0.36, 8.57)	0.485
Prior BSO	1.26 (0.52, 3.06)	0.612	1.00 (0.20, 4.88)	0.996
Genetics				
*BRCA1* or *BRCA2*	0.85 (0.27, 2.66)	0.776	0.83 (0.18, 3.79)	0.807
High penetrance	6.76 (2.09, 21.78)	0.001	0.50 (0.03, 9.78)	0.648
Moderate‐penetrance	1.95 (0.60, 6.33)	0.268	0.43 (0.02, 8.37)	0.579
VUS	0.92 (0.33, 2.56)	0.873	3.64 (0.11, 121.75)	0.471
No genetic testing	1.41 (0.68, 2.95)	0.359	1.92 (0.08, 47.42)	0.689
Preoperative MRI	0.43 (0.19, 0.99)	0.047	0.87 (0.13, 5.66)	0.887
Neoadjuvant chemotherapy	0.40 (0.14, 1.11)	0.078		
Index cancer		0.253	N/A	
Invasive ductal	Reference			
ILC or IMC	2.80 (1.04, 7.52)			
Other invasive	0.64 (0.06, 6.97)			
DCIS	1.72 (0.61, 4.88)			
Delayed PM/cancer treatment completed > 1 year before PM	0.24 (0.02, 2.53)			
Index cancer receptor status		0.393	N/A	
Estrogen receptor positive	Reference			
PR‐positive	1.34 (0.39, 4.62)			
HER2 neu positive	0.48 (0.05, 4.24)			
HER2 neu negative	0.82 (0.27, 2.53)			
ER positive versus not	2.77 (0.90, 8.56)	0.077	N/A	
PR positive versus not	2.18 (0.91, 5.24)	0.081	N/A	
Atypical lobular hyperplasia	1.93 (0.74, 5.06)	0.182	2.52 (0.37, 17.19)	0.345
Atypical ductal hyperplasia	2.72 (1.02, 7.25)	0.046	8.11 (0.97, 68.11)	0.054
History/presence of LCIS	4.09 (1.49, 11.23)	0.006	3.42 (0.48, 24.09)	0.218
Sentinel lymph node biopsy performed	3.87 (1.73, 8.68)	0.001	3.28 (0.64, 16.66)	0.153
Reconstruction	0.55 (0.28, 1.12)	0.098	1.60 (0.08, 31.82)	0.759
Body mass index	1.03 (0.98, 1.08)	0.188	1.03 (0.93, 1.15)	0.584
Obesity	0.54 (0.27, 1.08)	0.083	0.81 (0.18, 3.70)	0.783
Mantle field radiotherapy	2.70 (0.43, 17.04)	0.289	8.45 (0.08, 851.21)	0.365
Imaging to surgery (days)	1.00 (0.99, 1.00)	0.371	1.00 (0.99, 1.01)	0.748
Categories for imaging		0.623		0.704
1–60 days	Reference		Reference	
61–120 days	0.73 (0.28, 1.89)		0.77 (0.19, 3.20)	
121–180 days	0.64 (0.20, 2.10)		0.52 (0.10, 2.77)	
181–240 days	0.36 (0.08, 1.76)		0.60 (0.04, 8.55)	
241–300 days	1.92 (0.49, 7.58)		0.55 (0.04, 7.77)	
301–365 days	2.03 (0.10, 40.39)		4.57 (0.66, 31.87)	
MRI to surgery (days)	0.99 (0.98, 1.01)	0.389	1.01 (1.00, 1.01)	0.208
Categories for imaging		0.763		0.866
1–60 days	Reference		Reference	
61–120 days	1.27 (0.38, 4.25)		0.63 (0.13, 3.03)	
121––180 days	0.39 (0.03, 5.06)		0.72 (0.15, 3.48)	
181–240 days	0.30 (0.02, 3.87)		0.66 (0.05, 8.82)	
241–300 days	1.42 (0.09, 21.96)		2.15 (0.41, 11.38)	
301–365 days	3.04 (0.15, 62.91)		2.61 (0.48, 14.15)	

Abbreviations: BSO, bilateral salpino‐oophorectomy; DCIS, ductal carcinoma in‐situ; ER, estrogen receptor; HER2, human epidermal growth receptor 2; ILC, invasive lobular carcinoma; IMC, invasive mammary carcinoma; LCIS, lobular carcinoma in‐situ; MRI, magnetic resonance imaging; OR, odds ratio; PM, prophylactic mastectomy; PR, progesterone receptor; VUS, variant of unknown significance.

All analyses were conducted with SAS software, version 9.4 (SAS Institute Inc.).

## 3 RESULTS

### 3.1 Demographics and outcomes

The study population included 772 women, aged 20–79 years (mean 48.9 years) at the time of PM operation. Overall, there were 39 occult malignancies identified on pathologic examination of the prophylactic specimen: 17 cases of invasive cancers and 22 cases of DCIS. Of the 598 patients who underwent CPM, there were 14 cases of invasive cancer and 19 cases of DCIS detected incidentally, for an incidence of 5.5% (33/598) per PM. Of the 174 patients who underwent BPM resulting in a total of 348 PM specimens, there were 3 cases of invasive cancer and 3 cases of DCIS detected incidentally, for an incidence of 1.7% (6/348) of per PM. Additional patient demographics are displayed in Table 1.

Of the 17 invasive malignancies discovered incidentally, 16 were T1 lesions ranging from 0.15 to 1.6 cm. One patient underwent preoperative needle biopsy of a suspicious retroareolar finding which resulted as intraductal papilloma with atypia, however, a T2 papillary carcinoma was found on final pathologic assessment of the CPM specimen. Additional histopathologic characteristics of the PM specimens are presented in Table 4.

There were 86 patients who underwent prophylactic SLNB completed at the time of PM. There was one patient found to have a singular positive sentinel lymph node containing a micrometastatic focus. Of the 17 cases with an incidental finding of invasive carcinoma, 6 (4 CPM, 2 BPM) SLNB procedures were performed.

### 3.2 Variables of interest

**Figure 1:**
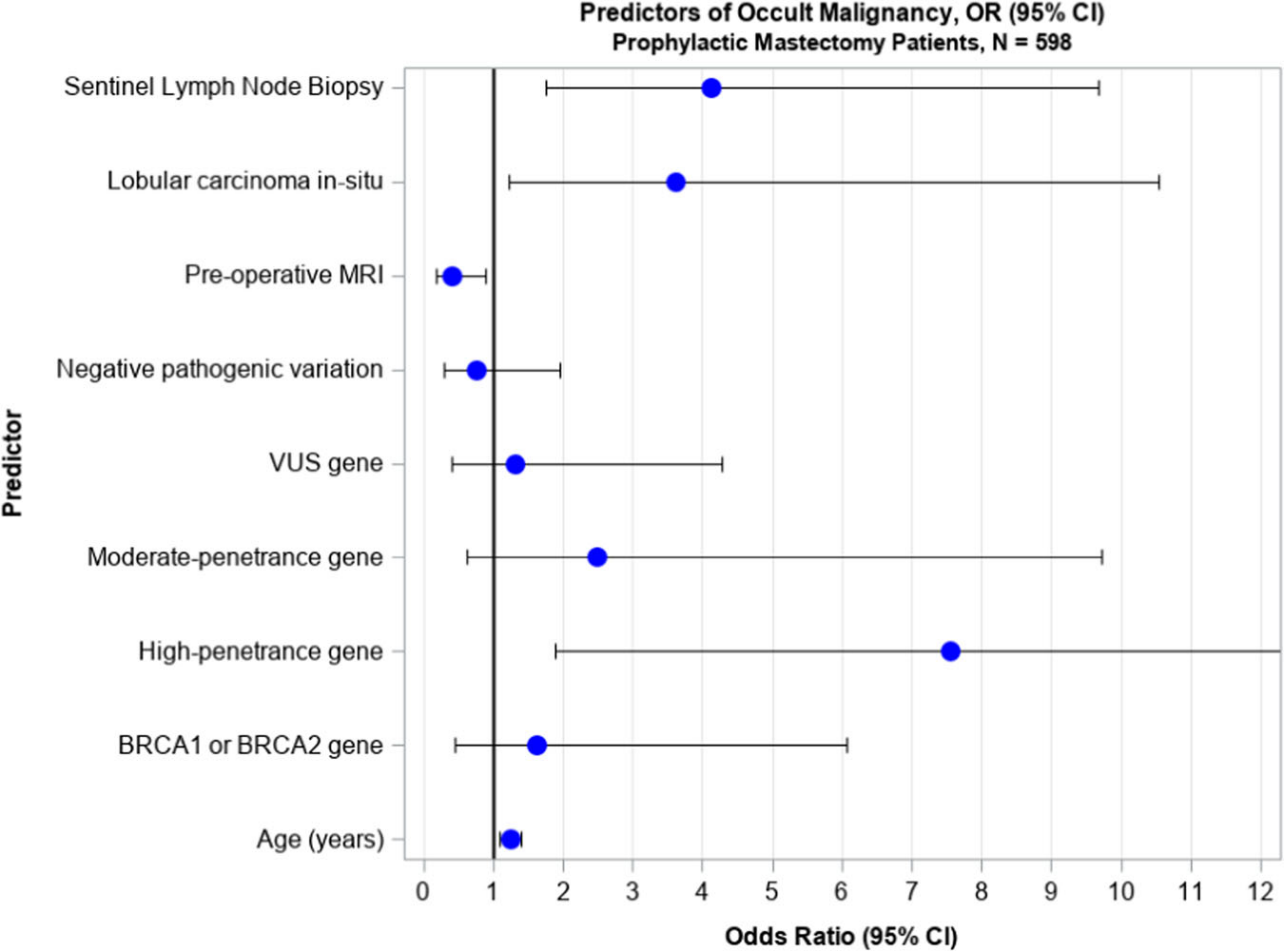
Individual factors associated with occult malignancy finding in contralateral prophylactic mastectomy patients, OR (95% CI), *N* = 598. CI, confidence interval; OR, odds ratio.

In univariate analysis (Table 2), the likelihood of OM among patients who underwent CPM increased with age (*p* = 0.008), a known non‐*BRCA* high‐penetrance pathogenic variant (*p* = 0.001), ADH within the prophylactic breast (*p* = 0.046), and the presence of LCIS in either the prophylactic or therapeutic mastectomy specimen (*p* = 0.006). The variables of age (*p* = 0.010), non‐*BRCA* high‐penetrance gene mutation (*p* = 0.004), and LCIS (*p* = 0.019) remained statistically significant on multivariate analysis (Table 3, Figure 1), however ADH did not retain significance. The prevalence of OM decreased in CPM patients who underwent preoperative MRI on both univariate (*p* = 0.047) and multivariate analysis (*p* = 0.043). There were no independent predictive factors associated with an OM among patients who underwent BPM.

**Table 3:** Individual factors associated with occult malignancy finding in contralateral prophylactic mastectomy patients, OR (95% CI), *N* = 598.

Predictor	OR (95% CI)	*p* Value
Age (years)	1.05 (1.01, 1.09)	0.010
Genetics^a^		
*BRCA1* or BRCA2	1.63 (0.44, 6.07)	0.463
High‐penetrance	7.55 (1.89, 30.06)	0.004
Moderate‐penetrance	2.48 (0.63, 9.72)	0.194
Variant of unknown significance	1.31 (0.40, 4.27)	0.652
Negative	0.76 (0.29, 1.95)	0.563
Preoperative magnetic resonance imaging	0.41 (0.18, 0.97)	0.043
History/presence of lobular carcinoma in‐situ	3.61 (1.23, 10.54)	0.019
Sentinel lymph node biopsy performed	4.13 (1.76, 9.67)	0.001

Abbreviations: CI, confidence interval; OR, odds ratio.

^a^ For each genotype, the reference is not having that particular genotype.

*C* = 0.791 (Good fitting model has *C* ≥ 0.7 (Hosmer & Lemeshow, 2013)^25^.

**Table 4:** Occult invasive malignancy characteristics

Bilateral or contral‐ateral mastectomy	Age	Histology	ER	PR	HER2	Histologic grade	Preop MRI	Genetics	Preoperative core needle biopsy	Sentinel node biopsy performed
Bilateral	23	Ductal	‐	‐	‐	3	Yes	*BRCA*1	No	Yes: negative
Bilateral	30	Ductal	+	‐	+	3	No	Negative	Yes: negative	No
Bilateral	55	Ductal	+	+	‐	2	Yes	Negative	Yes: negative	Yes: negative
Contralateral	63	Ductal	+	+	‐	1	No	Not performed	No	No
Contralateral	57	Ductal	+	+	‐	Not reported	No	Not performed	No	No
Contralateral	44	Ductal	+	+	‐	1	No	Negative	Yes: IDP	Yes: negative
Contralateral	66	Ductal	+	+	‐	1	Yes	Not performed	Yes: negative	No
Contralateral	50	Ductal	+	+	‐	1	No	*PALB2*	No	No
Contralateral	63	Lobular	+	+	‐	1	No	VUS *BARD1*	No	No
Contralateral	58	Ductal	+	+	‐	1	Yes	Not performed	Yes: negative	Yes: negative
Contralateral	69	Mammary	+	+	‐	1	No	Not performed	Yes: negative	No
Contralateral	43	Ductal	+	+	‐	3	No	Not performed	Yes: ADH	Yes: negative
Contralateral	56	Ductal	+	‐	‐	1	No	*TP53*	No	No
Contralateral	69	Papillary	+	‐	‐	1	No	Not performed	Yes: IDP	Yes: N1mi
Contralateral	42	Ductal	+	+	‐	2	No	*CHEK2*	No	No
Contralateral	58	Ductal	+	+	‐	2	No	*BRCA*2	No	No
Contralateral	51	Ductal	+	+	‐	2	No	*PALB2*	No	No

Abbreviations: ADH, atypical ductal hyperplasia; ER, estrogen receptor; HER2, human epidermal growth receptor 2; IDP, intraductal papilloma; PR, progesterone receptor; preop MRI, preoperative magnetic resonance imaging; VUS, variant of unknown significance.

A total of 605 (78.4%) patients underwent genetic testing with results available in the medical record. The BPM cohort had higher rates of *BRCA1/2*, high‐penetrance, and moderate‐penetrance genes. In contrast, the CPM cohort had higher rates of variant of unknown significance (VUS), negative genotype, and no genetic testing. With respect to known pathogenic germline variants, there were 88 patients with a deleterious *BRCA1* mutation (39 CPM, 49 BPM) and 77 patients with a deleterious *BRCA2* mutation (30 CPM, 37 BPM). Of the 241 *BRCA*‐positive specimens, there were 2 (0.8%) with findings of occult invasive ductal carcinoma and 4 (1.7%) with findings of DCIS. There were an additional 82 patients with deleterious mutations detected on multigene panel testing: 31 *CHEK2* (1 CPM with invasive OM, 1 CPM with DCIS), 22 *PALB2* (2 CPM with invasive OM), 17 *ATM* (1 CPM with DCIS), 7 *CDH*1, 3 *TP53* (1 CPM with invasive OM), and 2 *PTEN* (1 CPM with DCIS).

Among the CPM cohort there were 30 (5.0%) patients with LCIS of either breast, 5 of whom had an OM finding. Of the five cases with both LCIS and an incidental carcinoma, LCIS was present in the contralateral prophylactic specimen in two cases and reported in the therapeutic specimen in three cases. Among the BPM cohort there were 13 (7.5%) patients with LCIS of either breast, 1 of whom had an incidental invasive lobular carcinoma identified in the same breast.

Overall, 372 (48.2%) patients underwent breast MRI within 1 year of the date of surgery, 12 of whom were found to have an OM on final pathology. Within the BPM cohort, 81.0% (141/174) of patients had a preoperative MRI compared to 38.6% (231/598) of the CPM cohort. The median time between preoperative MRI and PM was 95.5 days for the overall study population, 94 days for those without OM findings, and 97 days for patients with occult disease. Among the six patients found to have an OM in a BPM specimen, one did not undergo MRI, four had negative or benign MRI findings, and one had a core needle biopsy demonstrating ADH. Of the 33 patients with OM detected in a CPM specimen, 26 did not undergo MRI, 4 had negative or benign MRI studies, 2 had benign core needle biopsies, and 1 proceeded to surgery before percutaneous biopsy of a breast‐imaging reporting and data system (BI‐RADS) 4 finding.

There were 10 (1.3%) documented deaths among the entire study population, none of whom had findings of OM. Of the 17 patients with invasive OM, there were no locoregional recurrences recorded in the electronic medical record.

## 4 DISCUSSION

Among 946 PM specimens from 772 patients, the overall rate of OM, including both invasive and in‐situ disease, was 4.1% (39/946) per PM specimen. Substratification of our study population into BPM and CPM cohorts denoted OM rates of 1.7% (6/348) and 5.5% (33/598) per PM specimen, respectively. Invasive disease was detected incidentally in 0.86% (3/348) and 2.3% (14/598) of BPM and CPM specimens, respectively. These values reflect those reported in earlier studies, with overall OM rates spanning from 3% to 10% and smaller percentages of occult invasive disease ranging from 1.0% to 2.6%.^12^, ^13^, ^14^, ^15^, ^16^, ^17^, ^18^, ^19^, ^20^

In the current study, 86 patients had SLNB procedures performed in conjunction with PM with one finding of nodal positivity. While axillary sampling was performed discretionarily at our institution, an association was demonstrated between prophylactic SLNB and OM findings in the CPM patient cohort. Reasons provided for selectively performing an SLNB in patients who were later found to have an OM finding included the presence of a high‐risk lesion on core needle biopsy of the prophylactic side, discordant biopsy results, or a BIRADS 4 finding not biopsied preoperatively due to either time constraints or patient preference.

While rates of OM and nodal positivity are similar among prior studies, the culmination of conclusions from the existing literature are variable. Burger et al. proposed that the benefits of contemporaneous SLNB compared to the risk of a secondary axillary surgery favor routine SLNB implementation.^26^ Bunting et al. advocated against both routine and selective approaches to SLNB in the prophylactic setting.^18^ Laronga et al. endorsed SLNB application for patients with contralateral locally advanced disease.^16^ Nagaraja et al. strongly recommended every patient undergo preoperative breast MRI before PM.^27^ Black et al. concluded that the costs and efficacy of MRI do not justify the routine performance of MRI in all PM patients. Murphy et al. supported the incorporation of intraoperative pathology to guide SLN surgery.^19^ Boughey et al. recommended consideration of SLN surgery for older women and patients with a history of LCIS or lobular cancer.^13^ Most recently, Wong et al. concluded that SLNB can be omitted in *BRCA*1/2 or *PALB2* carriers when preoperative MRI demonstrates BI‐RADS 1‐3 findings.^28^

In response to increasing national rates of CPM, the ASBrS issued a consensus statement addressing the utilization, risks, impact, and perspectives pertaining to CPM.^21^, ^29^ Shortly thereafter, the Society of Surgical Oncology Breast Disease Working Group provided an updated position statement regarding PM.^30^ Both panels, respectively, recommended against the routine performance of SLNB on the CPM side. The ASBrS authors proposed that axillary node sampling could be considered in the presence of suspicious lesions of the prophylactic breast not previously biopsied. Despite these guidelines, variation in practice patterns persists. Tasoulis et al. queried surgeons about practice patterns directed at their approach to SLNB use in the prophylactic setting. Of the 238 respondents, they found that 6.6% always perform SLNB, 26.3% sometimes perform SLNB, and 67.1% never perform SLNB in conjunction with PM.^22^

The ability to reliably identify sentinel nodes following mastectomy is impeded by disrupted or removed lymphatic channels. Consequently, patients with occult invasive disease who did not undergo upfront SLNB may be subjected to formal axillary dissection for cancer staging. In the absence of nodal involvement, the incorporation of sentinel node retrieval into PM cases can obviate the need for reoperation, axillary clearance, and corresponding treatment delays. Although generally considered to be a low‐risk operation, the morbidity profile of sentinel lymph node retrieval is not negligible and includes anaphylaxis related to isosulfan blue (0.1%), surgical site infection (1%–3%), hematoma (1.4%), reduced upper extremity range of motion (3.8%), lymphedema (5%–8%), seroma formation (6%–7%), sensory changes (8.6%–12%), and methylene blue dye induced skin necrosis (1.2%).^31^, ^32^, ^33^, ^34^, ^35^, ^36^ Those in support of routine axillary sampling at the time of PM maintain that the prospective advantages in the 1.0%–2.6% of cases with incidental invasive cancer findings^12^, ^13^, ^14^, ^15^, ^16^, ^17^, ^18^ outweigh the potential risks associated with SLNB. Conversely, advocates for prophylactic SLNB omission emphasize that the nodal positivity rate of 1.2%–1.9% in patients with OM^27^, ^37^ is less than the adverse surgical effect rates following sentinel node retrieval.^31^, ^32^ When axillary sampling is performed routinely with PM, the majority of patients are subjected to longer operative times, higher costs, and increased morbidity risks.

Of the 17 invasive occult malignancies in our study population, all but one were T1 lesions. Furthermore, with the exception of one triple negative OM, all incidentally detected invasive carcinomas were hormone receptor‐positive. Based on our data, genetic predisposition was not a risk factor for OM in the BPM cohort. While previous articles have reported on the lack of association between *BRCA* mutation carriers and incidental cancer,^20^, ^38^, ^39^ our study is one of the first to examine additional high‐penetrance genes. Although we found patients with a known high‐penetrance gene mutation undergoing CPM to be an increased risk of OM, pathogenic germline variant status alone should not be an indication for prophylactic axillary sampling.

The role of breast MRI as a noninvasive diagnostic modality to identify occult disease preoperatively is not well‐defined. McLaughlin et al. reported a negative predictive value of 98% when MRI was obtained before PM, and concluded that patients with an unremarkable preoperative MRI of the prophylactic breast could selectively forgo SLNB.^15^ Conversely, Erdahl et al. found no correlation between preoperative MRI results and OM findings.^38^ In our CPM study population, preoperative breast MRI within 1 year of surgery correlated with a lower risk of OM on both univariate and multivariate analysis. There were no significant differences between incidental cancer detection and the time lapsed from preoperative MRI to surgery. While breast MRI is not a requisite for every patient pursuing PM, the utility of MRI could be of value for patients in the older decades of life with additional risk factors, such as LCIS or a known high‐penetrance gene, undergoing CPM.

The incidence of OM in our study population was low, though not zero. Historically, the opportunity to reliably retrieve sentinel lymph nodes was relinquished following mastectomy, however a novel nonradioactive tracer has recently emerged as a potential proxy to prophylactic SLNB. The role of superparamagnetic iron oxide nanoparticles (SPIO) for the purposes of delayed axillary staging in is an area of active investigation. Compared to technetium‐99m, which is limited by a half‐life of 6 h, SPIO can be detected up to 6 weeks after injection.^40^ The aim of the ongoing SentiNOT 2.0 phase 3 multi‐institutional randomized controlled trial (NCT04722692) is to elucidate the efficacy of SPIO injection and delayed SLNB. In addition to the inclusion of patients diagnosed with a pre‐invasive or discordant lesion preoperatively, the protocol includes patients undergoing PM. Pending the results of the SentiNOT 2.0 study, the use of SPIO in the setting of PM could be a consideration as an intermediary option to routine SLNB in future practice.

## 5 CONCLUSIONS

In recent years, there has been heightened interest in reducing low‐value cancer care, including research specifically directed toward overtreatment of the axilla. The results of our study do not support the routine use of intraoperative lymphatic mapping and SLNB in the setting of bilateral or contralateral PM. This investigation was limited by its single‐institutional, retrospective design and prospective assessment is forthcoming. Based on the current study, selective implementation of axillary lymph node sampling in the setting of PM could be considered for patients with indeterminate radiographic findings and discordant preoperative biopsy results, particularly in patients undergoing CPM with a non‐*BRCA* high‐penetrance pathogenic mutation or known LCIS.

## CONFLICT OF INTEREST

The authors declare no conflict of interest.

## SYNOPSIS

Occult invasive breast cancer was identified in 1.8% of bilateral and contralateral prophylactic mastectomies. Factors associated with increased risk of occult malignancy included age, LCIS, and non‐*BRCA* high‐penetrance gene. Prophylactic sentinel node biopsy should not be performed routinely.

## Data Availability

The data that support the findings of this study are available from the corresponding author upon reasonable request.

## References

[1] Baskin AS , Wang T , Bredbeck BC , Sinco BR , Berlin NL , Dossett LA . Trends in contralateral prophylactic mastectomy utilization for small unilateral breast cancer. J Surg Res. 2021;262:71‐84. 10.1016/j.jss.2020.12.057 33548676 PMC8043987

[2] Panchal H , Pilewskie ML , Sheckter CC , et al. National trends in contralateral prophylactic mastectomy in women with locally advanced breast cancer. J Surg Oncol. 2019;119(1):79‐87. 10.1002/jso.25315 30480805 PMC6327316

[3] Wang T , Baskin AS , Dossett LA . Deimplementation of the choosing wisely recommendations for low‐value breast cancer surgery: a systematic review. JAMA Surg. 2020;155:759‐770. 10.1001/jamasurg.2020.0322 32492121 PMC10185302

[4] Chung A , Huynh K , Lawrence C , Sim MS , Giuliano A . Comparison of patient characteristics and outcomes of contralateral prophylactic mastectomy and unilateral total mastectomy in breast cancer patients. Ann Surg Oncol. 2012;19:2600‐2606. 10.1245/S10434-012-2299-1 22396004

[5] King TA , Sakr R , Patil S , et al. Clinical management factors contribute to the decision for contralateral prophylactic mastectomy. J Clin Oncol. 2011;29(16):2158‐2164. 10.1200/JCO.2010.29.4041 21464413

[6] Kurian AW , Ward KC , Abrahamse P , et al. Association of germline genetic testing results with locoregional and systemic therapy in patients with breast cancer. JAMA Oncol. 2020;6(4):e196400. 10.1001/jamaoncol.2019.6400 32027353 PMC7042883

[7] Kurian AW , Li Y , Hamilton AS , et al. Gaps in incorporating germline genetic testing into treatment decision‐making for early‐stage breast cancer. J Clin Oncol. 2017;35:2232‐2239. 10.1200/JCO 28402748 PMC5501363

[8] Santosa KB , Oliver JD , Momoh AO . Contralateral prophylactic mastectomy and implications for breast reconstruction. Gland Surg. 2021;10(1):498‐506. 10.21037/gs.2020.03.15 33634008 PMC7882322

[9] Hoskin TL , Hieken TJ , Degnim AC , Jakub JW , Jacobson SR , Boughey JC . Use of immediate breast reconstruction and choice for contralateral prophylactic mastectomy. Surgery. 2016;159(4):1199‐1209. 10.1016/j.surg.2015.11.001 26704783

[10] Agarwal S , Kidwell KM , Kraft CT , et al. Defining the relationship between patient decisions to undergo breast reconstruction and contralateral prophylactic mastectomy. Plast Reconstr Surg. 2015;135(3):661‐670. 10.1097/PRS.0000000000001044 25719688 PMC4822506

[11] Houssami N , Turner RM , Morrow M . Meta‐analysis of pre‐operative magnetic resonance imaging (MRI) and surgical treatment for breast cancer. Breast Cancer Res Treat. 2017;165(2):273‐283. 10.1007/s10549-017-4324-3 28589366 PMC5580248

[12] King TA , Ganaraj A , Fey JV , et al. Cytokeratin‐positive cells in sentinel lymph nodes in breast cancer are not random events: experience in patients undergoing prophylactic mastectomy. Cancer. 2004;101(5):926‐933. 10.1002/cncr.20517 15329899

[13] Boughey JC , Khakpour N , Meric‐Bernstam F , et al. Selective use of sentinel lymph node surgery during prophylactic mastectomy. Cancer. 2006;107(7):1440‐1447. 10.1002/cncr.22176 16955504

[14] Black D , Specht M , Lee JM , et al. Detecting occult malignancy in prophylactic mastectomy: preoperative MRI versus sentinel lymph node biopsy. Ann Surg Oncol. 2007;14(9):2477‐2484. 10.1245/s10434-007-9356-1 17587091

[15] McLaughlin SA , Stempel M , Morris EA , Liberman L , King TA . Can magnetic resonance imaging be used to select patients for sentinel lymph node biopsy in prophylactic mastectomy. Cancer. 2008;112(6):1214‐1221. 10.1002/cncr.23298 18257089

[16] Laronga C , Lee MC , McGuire KP , et al. Indications for sentinel lymph node biopsy in the setting of prophylactic mastectomy. J Am Coll Surg. 2009;209(6):746‐752. 10.1016/j.jamcollsurg.2009.08.010 19959044

[17] Czyszczon IA , Roland L , Sahoo S . Routine prophylactic sentinel lymph node biopsy is not indicated in women undergoing prophylactic mastectomy. J Surg Oncol. 2012;105(7):650‐654. 10.1002/jso.23018 22213101

[18] Bunting PW , Cyr AE , Gao F , Margenthaler JA . Sentinel lymph node biopsy during prophylactic mastectomy: is there a role? J Surg Oncol. 2014;109(8):747‐750. 10.1002/jso.23575 24535940

[19] Murphy BL , Glasgow AE , Keeney GL , Habermann EB , Boughey JC . Selective use of sentinel lymph node surgery in patients undergoing prophylactic mastectomy using intraoperative pathology. Ann Surg Oncol. 2017;24(10):3032‐3037. 10.1245/s10434-017-5925-0 28766201

[20] Mattos D , Gfrerer L , Ling IT , et al. Occult histopathology and its predictors in contralateral and bilateral prophylactic mastectomies. Ann Surg Oncol. 2016;23(3):767‐775. 10.1245/s10434-015-4896-2 26577123

[21] Boughey JC , Attai DJ , Chen SL , et al. Contralateral prophylactic mastectomy consensus statement from the American Society of Breast Surgeons: additional considerations and a framework for shared decision making. Ann Surg Oncol. 2016;23(10):3106‐3111. 10.1245/s10434-016-5408-8 27469118 PMC4999472

[22] Tasoulis MK , Hughes T , Babiera G , Chagpar AB . Sentinel lymph node biopsy in low risk settings. Am J Surg. 2017;214(3):489‐494. 10.1016/j.amjsurg.2017.03.006 28335989

[23] Firth D . Bias reduction of maximum likelihood estimates. Biometrika. 1993;80:27‐38. 10.2307/2336755

[24] Akaike H . A new look at the statistical model identification. IEEE Trans Autom Control. 1974;19(6):716‐723. 10.1007/978-1-4612-1694-0_16

[25] Hosmer DW , Lemeshow S , Sturdivant RX . Applied Logistic Regression. 3rd ed. John Wiley & Sons; 2013.

[26] Burger A , Thurtle D , Owen S , et al. Sentinel lymph node biopsy for risk‐reducing mastectomy. Breast J. 2013;19(5):529‐532. 10.1111/tbj.12157 23865803

[27] Nagaraja V , Edirimanne S , Eslick GD . Is sentinel lymph node biopsy necessary in patients undergoing prophylactic mastectomy? a systematic review and meta‐analysis. Breast J. 2016;22(2):158‐165. 10.1111/tbj.12549 26748493

[28] Wong SM , Ferroum A , Apostolova C , et al. Incidence of occult breast cancer in carriers of BRCA1/2 or other high‐penetrance pathogenic variants undergoing prophylactic mastectomy: when is sentinel lymph node biopsy indicated? Ann Surg Oncol . Published online May 26, 2022. 10.1245/S10434-022-11916-3 35616744

[29] Boughey JC , Attai DJ , Chen SL , et al. Contralateral prophylactic mastectomy (CPM) consensus statement from the American Society of Breast Surgeons: data on CPM outcomes and risks. Ann Surg Oncol. 2016;23(10):3100‐3105. 10.1245/s10434-016-5443-5 27469117 PMC4999465

[30] Hunt KK , Euhus DM , Boughey JC , et al. Society of Surgical Oncology Breast Disease Working Group Statement on Prophylactic (risk‐reducing) Mastectomy. Ann Surg Oncol. 2017;24:375‐397. 10.1245/S10434-016-5688-Z 27933411

[31] Wilke LG , McCall LM , Posther KE , et al. Surgical complications associated with sentinel lymph node biopsy: results from a prospective international cooperative group trial. Ann Surg Oncol. 2006;13(4):491‐500. 10.1245/ASO.2006.05.013 16514477

[32] Lucci A , McCall LM , Beitsch PD , et al. Surgical complications associated with sentinel lymph node dissection (SLND) plus axillary lymph node dissection compared with SLND alone in the American College of Surgeons Oncology Group trial Z0011. J Clin Oncol. 2007;25(24):3657‐3663. 10.1200/JCO.2006.07.4062 17485711

[33] Mansel RE , Fallowfield L , Kissin M , et al. Randomized multicenter trial of sentinel node biopsy versus standard axillary treatment in operable breast cancer: the ALMANAC trial. J Natl Cancer Inst. 2006;98(9):599‐609. 10.1093/jnci/djj158 16670385

[34] McLaughlin SA , Wright MJ , Morris KT , et al. Prevalence of lymphedema in women with breast cancer 5 years after sentinel lymph node biopsy or axillary dissection: objective measurements. J Clin Oncol. 2008;26(32):5213‐5219. 10.1200/JCO.2008.16.3725 18838709 PMC2652091

[35] Ashikaga T , Krag DN , Land SR , et al. Morbidity results from the NSABP B‐32 trial comparing sentinel lymph node dissection versus axillary dissection. J Surg Oncol. 2010;102(2):111‐118. 10.1002/jso.21535 20648579 PMC3072246

[36] Zakaria S , Hoskin TL , Degnim AC . Safety and technical success of methylene blue dye for lymphatic mapping in breast cancer. Am J Surg. 2008;196(2):228‐233. 10.1016/j.amjsurg.2007.08.060 18367146

[37] Zhou WBin , Liu XA , Dai JC , Wang S . Meta‐analysis of sentinel lymph node biopsy at the time of prophylactic mastectomy of the breast. Can J Surg. 2011;54(5):300‐306. 10.1503/cjs.006010 21651834 PMC3195666

[38] Erdahl LM , Boughey JC , Hoskin TL , Degnim AC , Hieken TJ . Contralateral prophylactic mastectomy: factors predictive of occult malignancy or high‐risk lesion and the impact of MRI and genetic testing. Ann Surg Oncol. 2016;23(1):72‐77. 10.1245/s10434-015-4660-7 26065870

[39] Câmara S , Pereira D , André S , et al. The use of sentinel lymph node biopsy in BRCA1/2 mutation carriers undergoing prophylactic mastectomy: s retrospective consecutive case‐series study. Int J Breast Cancer. 2018;2018:1426369. 10.1155/2018/1426369 29507815 PMC5817815

[40] Karakatsanis A , Hersi A‐F , Pistiolis L , et al. Effect of preoperative injection of superparamagnetic iron oxide particles on rates of sentinel lymph node dissection in women undergoing surgery for ductal carcinoma in situ (SentiNot study). Br J Surg. 2019;106(6):720‐728. 10.1002/bjs.11110 30839104

